# Case Report: Glaucoma in an Infant With Retinopathy of Prematurity

**DOI:** 10.3389/fped.2021.786327

**Published:** 2021-12-16

**Authors:** Tamara Lee Lenis, Nahomy Ledesma Vicioso, Varun Reddy, Kyle D Kovacs, Sarah H Van Tassel, Anton Orlin

**Affiliations:** Department of Ophthalmology, Weill Cornell Medicine, New York Presbyterian Hospital, New York, NY, United States

**Keywords:** retinopathy of prematurity (ROP), glaucoma, anti-VEGF (vascular endothelial growth factor), laser retinal photocoagulation, myopia, case report

## Abstract

Retinopathy of prematurity (ROP) is a leading cause of childhood blindness that occurs due to incomplete development of retinal blood vessels in preterm infants. Glaucoma is an ocular comorbidity in some patients with ROP, and it may be associated with immature anterior chamber development, ROP itself, or the treatment for ROP. There have been a few reports of narrow-angle glaucoma after laser treatment for ROP. In this case report, we describe the course of a female infant born at 24 weeks and 5 days of gestational age with treatment-requiring ROP treated with laser photocoagulation who subsequently developed very elevated intraocular pressure and shallow anterior chambers without pupillary block. The patient required bilateral *ab externo* trabeculotomy for elevated intraocular pressure, which normalized after the procedure. The patient has remained stable at the last follow-up at 51 weeks postmenstrual age. Differing from previous glaucoma presentations in this setting, we illustrate a case of elevated intraocular pressure and anterior chamber narrowing after laser therapy without pupillary block or synechiae. The possible multifactorial etiology of glaucoma in this patient, including incomplete angle development, ischemia, and laser treatment, highlight the need for glaucoma screening in patients with ROP, both in the short and long term.

## Introduction

Retinopathy of prematurity (ROP) is a disorder of incomplete development of retinal blood vessels in preterm infants (1). Although titration of supplemental oxygen, which has been shown to decrease ischemic drive, has decreased the incidence of ROP, this condition remains a leading cause of childhood blindness in the United States (2) and throughout the world (3). The main goals of treatment are to optimize long-term visual outcomes by preventing complications of ROP such as retinal detachment (4). Laser photocoagulation has replaced cryotherapy as the standard of care for treatment-requiring ROP (5, 6), while intravitreal antivascular endothelial growth factor (anti-VEGF) injections are also used with similar efficacy (5), particularly in posterior ROP (6).

Treatment of ROP has known potential complications, including cataract, inflammation, vitreous hemorrhage, choroidal detachment, retinal detachment, and glaucoma (7, 8). Patients with ROP are at increased risk for glaucoma, which can be attributed to retrolental tissue pushing of the lens–iris diaphragm forward (9, 10), as well as pupillary block or neovascularization (11, 12).

The Early Treatment for Retinopathy of Prematurity (ETROP) trial found a 1.67% (12/718) prevalence of glaucoma at 6 years old (9, 10), with 11 out of these 12 children having received laser therapy for high-risk pre-threshold ROP (13). There are few cases reported of narrow angle or angle-closure glaucoma following laser for ROP (14–16).

In this case report, we describe the course of an ex-24-week-old female infant who developed very elevated intraocular pressure and shallow anterior chambers without pupillary block at postmenstrual age of 38 weeks following laser treatment in both eyes for type 1 ROP (zone 2 stage 3 with plus disease). Examination was remarkable for corneal haze that precluded gonioscopy and shallow anterior chambers without pupillary block or neovascularization. Genetic testing for the most common causes of congenital glaucoma was negative. There was significant improvement in the anterior chamber (AC) depth with cycloplegia; however, the patient ultimately required bilateral *ab externo* trabeculotomy for adequate intraocular pressure control. This case highlights that glaucoma in the setting of ROP may be multifactorial and should be regularly screened for in any at-risk infant.

## Case

**Figure 1:**
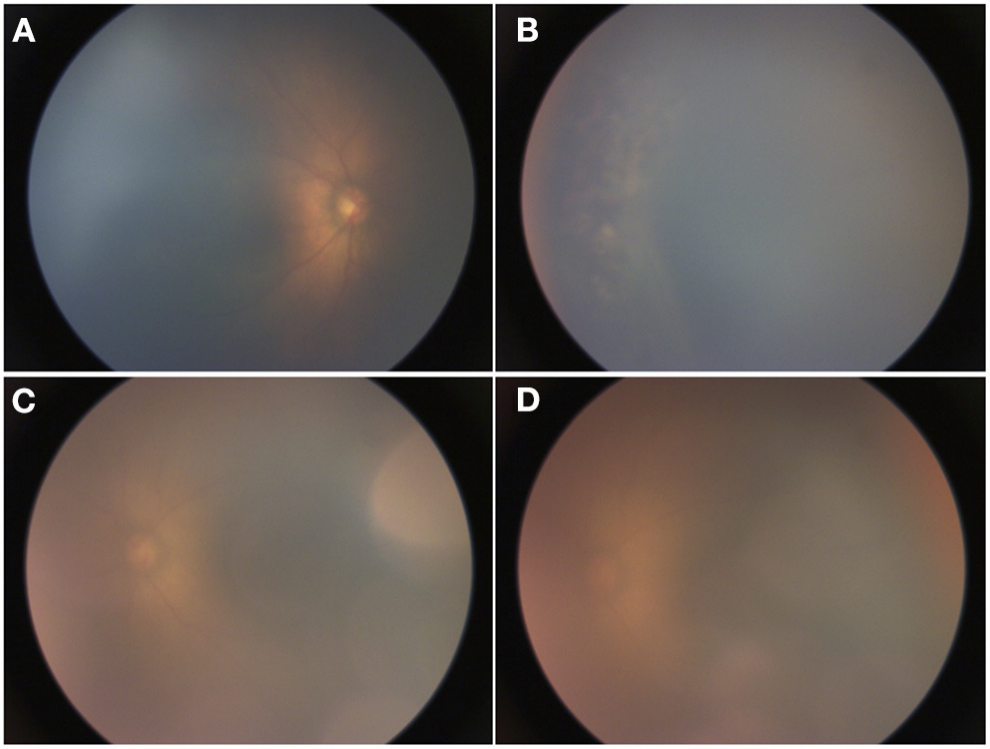
RetCam images taken at 37 weeks of postmenstrual age (PMA). **(A)** Right eye with zone 2, stage 3 retinopathy of prematurity (ROP), **(B)** limited view of laser scars in the periphery of the right eye, and **(C,D)** left eye with zone 2, stage 3 ROP.

A 450-g female infant was born at 24 weeks and 5 days of gestational age by normal spontaneous vaginal delivery at an outside hospital and transferred to our institution at a postmenstrual age (PMA) of 33 weeks and 2 days for patent ductus arteriosus ligation. At our institution, she underwent an initial ROP screening, which showed stage 0 immature vessels in zone 2 of both eyes. There were clear views of the fundus in both eyes. The patient was then transferred back to the home referring hospital where she underwent two sessions of diode laser in both eyes at 36 and 37 weeks of PMA for treatment-requiring ROP. Due to concern for inadequate laser and poor fundus view with corneal haze, she was re-referred to our institution. Upon reevaluation, she was noted to have cloudy corneas, intraocular pressure (IOP) of 40–45 mmHg, and persistent zone 2, stage 3 ROP, with peripheral laser scars in both eyes (Figure 1).

**Figure 2:**
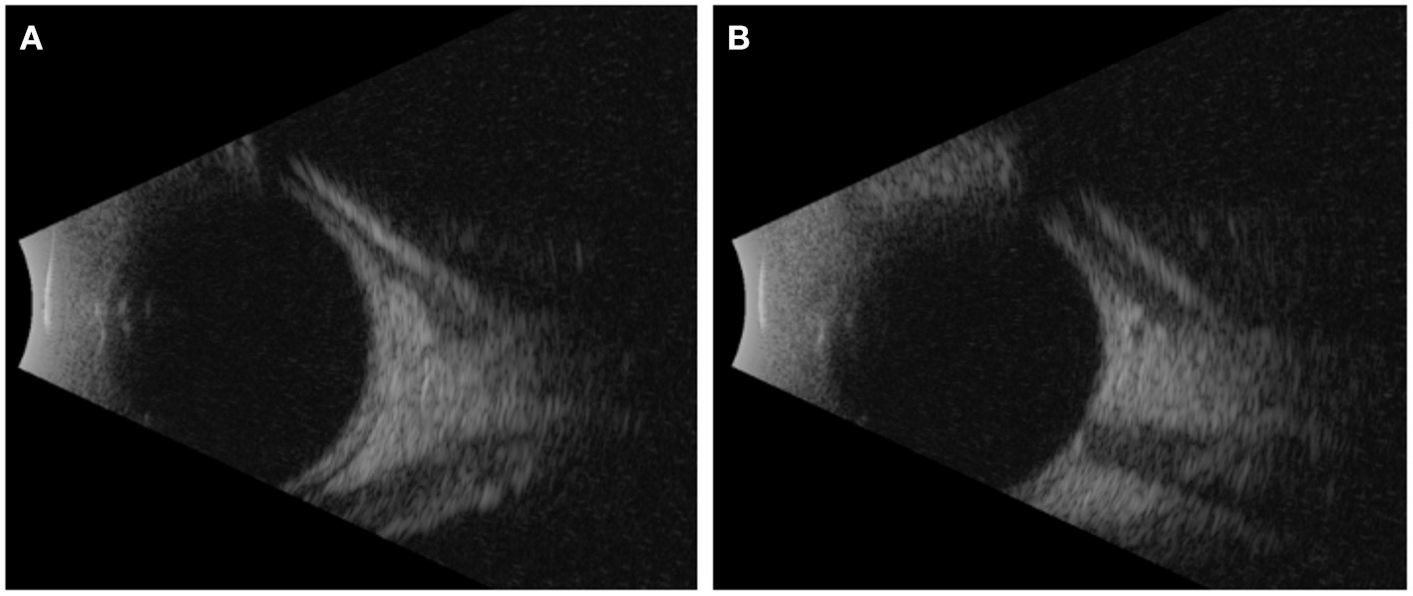
B-scan ultrasound images taken at 40 weeks of PMA. **(A)** Right eye and **(B)** left eye.

**Figure 3:**
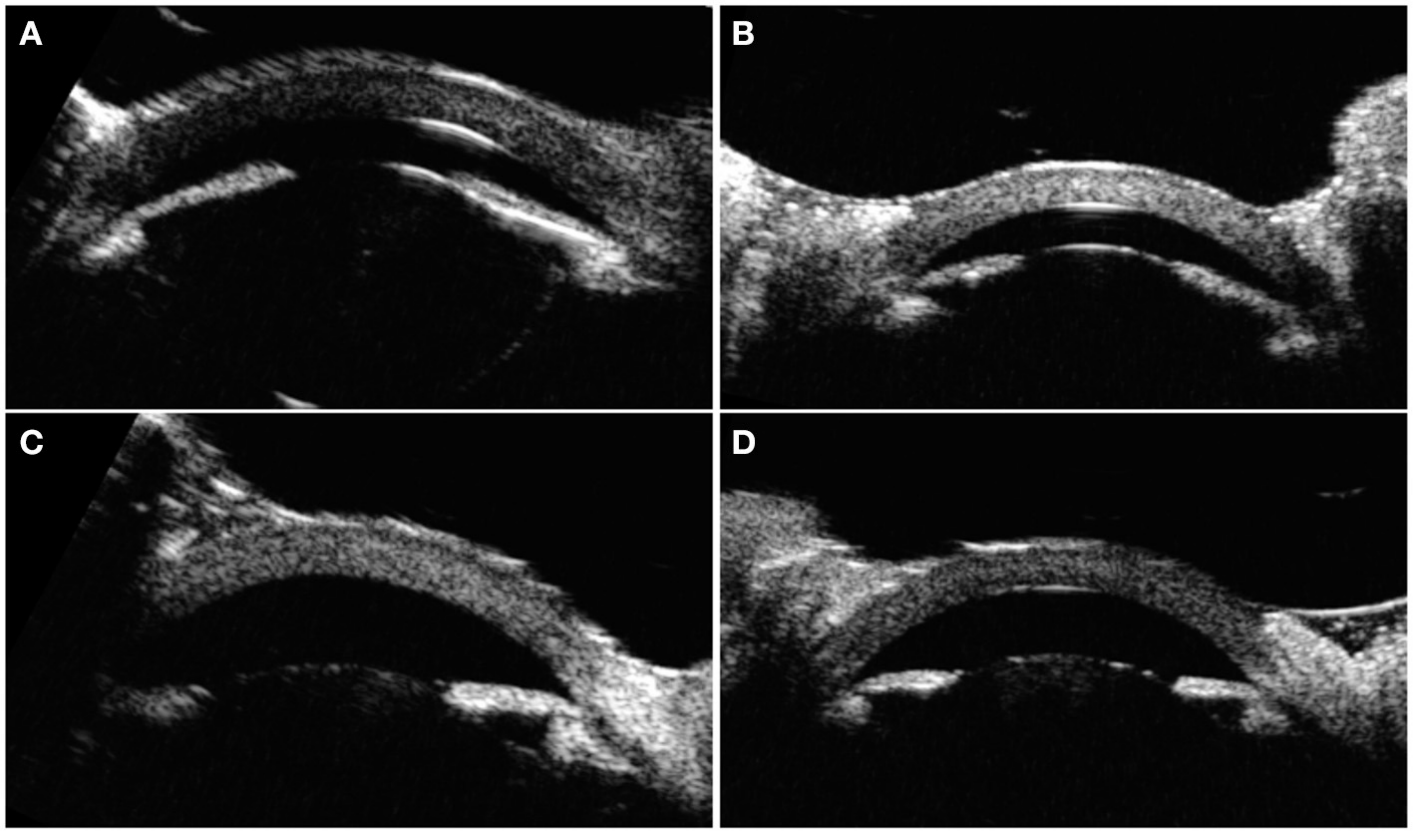
Ultrasound biomicroscopy images taken at 40 and 41 weeks of PMA. **(A)** Right eye before cyclopegia. **(B)** Left eye before cyclopegia. **(C)** Right eye after cyclopegia. **(D)** Left eye after cyclopegia.

On exam and diagnostic imaging with B-scan ultrasound, there was no rubeosis, and no choroidal or retinal detachments were noted (Figure 2). Clinical examination and ultrasound biomicroscopy (UBM) revealed significant anterior chamber shallowing without pupillary block or synechiae, which improved significantly with topical cycloplegia with cyclopentolate 0.5% (Figure 3). With oral acetazolamide and topical IOP-lowering eyedrops, her pressures improved but were still elevated to the 30- to 35-mmHg range, and the corneal clouding still prevented adequate gonioscopic visualization of angle anatomy. Fundus examination revealed persistent stage 3 ROP with inadequate laser, particularly nasally; however, the view was still somewhat limited for additional laser photocoagulation (Figure 1). She underwent intravitreal injection of bevacizumab 0.625 mg in both eyes at 39 weeks of PMA. In both eyes, regression of ROP after laser and intravitreal bevacizumab was noted by 41 weeks of PMA (2 weeks after injections). Despite moderate improvement in the anterior chamber depth (Figure 3) and IOP, medical treatments were ultimately determined to be inadequate, and the patient required *ab externo* trabeculotomy in the right eye at 42 weeks of PMA and left eye at 44 weeks of PMA. With parental consent, genetic screening for congenital glaucoma through the Prevention Genetics Glaucoma Panel was obtained and found to be negative.

**Figure 4:**
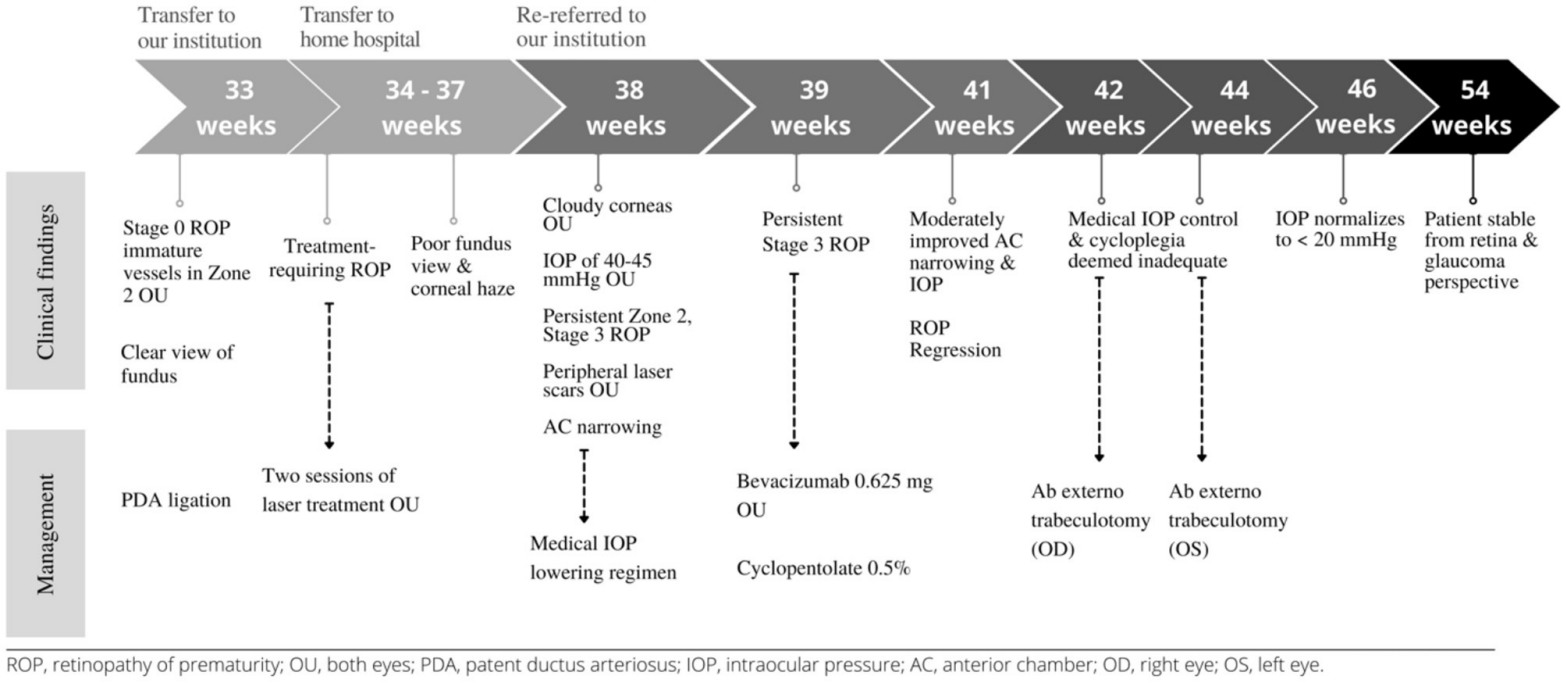
Timeline of clinical findings and management of patient.

The intraocular pressures normalized to under 20 mmHg by postoperative week 2 after trabeculotomy in both eyes. The patient has remained stable from both a retina and glaucoma perspective at the last follow-up at 51 weeks of PMA in the office. Of note, cycloplegic refraction revealed −8.00 and −2.00 D myopia in the right and left eyes, respectively, for which she was prescribed spectacle correction. The timeline of clinical findings and management of this patient are presented (Figure 4). Written informed consent was obtained from the legal guardian of the minor for the publication of any potentially identifiable images or data included in this article.

## Discussion

The pathogenesis of glaucoma in ROP has various proposed mechanisms including incomplete development of the anterior segment and secondary angle closure from ROP or the treatment of ROP. Given the presentation and exam findings of our patient, it is possible that underlying incomplete development of her anterior segment structure, as well as ROP, may have put her at increased risk for glaucoma that was further exacerbated by ROP laser treatment though no effusions or clear sequelae of this were observed.

There are few cases in the literature describing glaucoma in the short-term postoperative period after ROP laser (14–16). The cases requiring surgical intervention presented with shallow or flat AC depth and had documented posterior synechiae with pupillary block in infants with gestational ages ranging from 24 to 29 weeks (14). A report from Australia described the case of a male infant of 24 weeks of gestational age receiving laser therapy for stage 2 zone 3 ROP who was later found to have pupillary block and angle closure in the right eye as well as anatomic narrow angle in the left eye (15). A third report described a female infant of 25 weeks of gestational age who received laser photocoagulation for ROP in both eyes (stage 2, zone 2 with plus disease), after which she developed total posterior synechiae and shallow ACs (16).

In our case, the initial treatment with topical cycloplegia and IOP-lowering topical and oral medications led to deepening of the anterior chamber as well as moderate, though ultimately insufficient, improvement of the IOP. Normalization of IOP was achieved after *ab externo* trabeculotomy in both eyes. This points to a possible mixed mechanism of glaucoma in infants with ROP after laser therapy: (1) abnormal angle anatomy from prematurity or underlying genetic predisposition, and (2) ciliary body rotation, possibly exacerbated by laser. In contrast to the previously described cases in the literature (14–16), our case illustrates a presentation of elevated IOP and anterior chamber narrowing after laser therapy without pupillary block or synechiae.

Prematurity itself introduces some structural risks that may contribute to glaucoma. Development of the trabecular meshwork is not complete until late in gestation, after at least 25 weeks, which makes the angle anatomy susceptible to many factors that can potentially influence risk of angle-closure glaucoma (17). Other predisposing anterior segment abnormalities observed in premature children, with and without ROP, include steep corneal curvature, decreased anterior chamber depth, anteriorly displaced iris planes, and increased lens thickness (10, 18–22). Eyes with ROP not requiring treatment as well as ROP-treated eyes have been found to have narrower anterior chamber angles (ACA) than full-term children (22) or preterm monitored eyes (23). These changes may also explain the high prevalence and magnitude of myopia in preterm children (24), and the even higher apparent risk in those with laser-treated ROP (21). It is notable that our patient demonstrated a high degree of myopia in right eye and a moderate degree of myopia in the left eye at just 11 months of PMA.

Premature infants with ROP likely acquire other additional pathologic features that put them at additional risk of glaucoma. The anterior segment changes observed in premature infants with ROP could be further exacerbated by an arrested state of retinal development, such that the local growth signals involved in anterior segment development are altered (24). Additionally, laser treatment is considered to contribute significantly to the development of narrow angles and anterior segment defects in children with ROP (23, 24). More specifically, laser-induced mechanical changes may impair development of the angle by affecting the posterior ciliary nerve, artery, or pars plana (23). While anterior chamber narrowing without closure has been previously described after laser for other conditions, the laser-induced anterior shifting and inward rotation of the ciliary body is typically short lived in older children and adults (25). In a case series of secondary glaucoma in children with ROP, all of the patients had shallow anterior chambers before the onset of glaucoma symptoms, so these patients might have had an initial anatomic compromise of the aqueous outflow subsequently exacerbated by other factors leading to high intraocular pressures (26). Likewise, in another study, shallow anterior chambers were noted to be a feature of most stage 3 ROP eyes, as a result of arrested development of the anterior segment, but with notable higher and more significant prevalence in laser-treated eyes (27). Given the presentation and exam findings of our patient, it is possible that underlying developmental anterior segment abnormalities, as well as ROP, may have put her at increased risk for glaucoma that was further exacerbated after ROP laser treatment. A potential future avenue for research would be to investigate the use of anti-VEGF injections as an alternative to laser in infants with significant narrow-angle glaucoma or anterior segment narrowing.

Laser treatment may induce structural anterior segment changes in infants with ROP and increase their vulnerability to complications, such as secondary glaucoma. Careful anterior segment examination in ROP infants should be undertaken to exclude glaucoma risk factors, such as shallow anterior chambers, rubeosis, and ocular hypertension. In particular, shallow anterior chambers are challenging to assess in premature eyes and can be overlooked unless accompanied by more obvious clinical signs of glaucoma like corneal clouding, enlarged corneal diameters, and blepharospasm (14). Glaucoma poses a vision-threatening risk in infants with ROP, due to premature development of the eye, as well as direct consequences of ROP and ROP treatment. While congenital glaucoma is a possible etiology in any infant with elevated intraocular pressure and cloudy corneas, this case highlights other contributory factors, such as prematurity itself, as well as laser for ROP, that should be considered.

## Data Availability Statement

The original contributions presented in the study are included in the article/supplementary material, further inquiries can be directed to the corresponding author/s.

## Ethics Statement

Written informed consent was obtained from the minors' legal guardian for the publication of any potentially identifiable images or data included in this article.

## Author Contributions

TL, NL, and AO contributed to the conception and design of the report. TL, AO, VR, KK, and SV participated in the patient's care. TL and NL wrote the first draft of the manuscript. VR, KK, SV, and AO wrote sections of the manuscript. All authors contributed to manuscript revision, read, and approved the submitted version.

## Conflict of Interest

The authors declare that the research was conducted in the absence of any commercial or financial relationships that could be construed as a potential conflict of interest. The handling Editor declared a past collaboration with one of the authors TL.

## Publisher's Note

All claims expressed in this article are solely those of the authors and do not necessarily represent those of their affiliated organizations, or those of the publisher, the editors and the reviewers. Any product that may be evaluated in this article, or claim that may be made by its manufacturer, is not guaranteed or endorsed by the publisher.
